# MEMS based highly sensitive dual FET gas sensor using graphene decorated Pd-Ag alloy nanoparticles for H_2_ detection

**DOI:** 10.1038/s41598-018-24324-z

**Published:** 2018-04-12

**Authors:** Bharat Sharma, Jung-Sik Kim

**Affiliations:** Department of Materials Science and Engineering, University of Seoul, Seoul, 02504 Korea

## Abstract

A low power, dual-gate field-effect transistor (FET) hydrogen gas sensor with graphene decorated Pd-Ag for hydrogen sensing applications was developed. The FET hydrogen sensor was integrated with a graphene-Pd-Ag-gate FET (GPA-FET) as hydrogen sensor coupled with Pt-gate FET as a reference sensor on a single sensor platform. The sensing gate electrode was modified with graphene by an e-spray technique followed by Pd-Ag DC/MF sputtering. Morphological and structural properties were studied by FESEM and Raman spectroscopy. FEM simulations were performed to confirm the uniform temperature control at the sensing gate electrode. The GPA-FET showed a high sensing response to hydrogen gas at the temperature of 25~254.5 °C. The as-proposed FET H_2_ sensor showed the fast response time and recovery time of 16 s, 14 s, respectively at the operating temperature of 245 °C. The variation in drain current was positively related with increased working temperature and hydrogen concentration. The proposed dual-gate FET gas sensor in this study has potential applications in various fields, such as electronic noses and automobiles, owing to its low-power consumption, easy integration, good thermal stability and enhanced hydrogen sensing properties.

## Introduction

Hydrogen (H_2_) is viewed as the best clean energy carriers which are the decisive fossil fuel candidate, with low minimum ignition energy and high heat of combustion^1,2^. Recently, hydrogen has been extensively used in various applications, such as fuel cells, power generators, automobiles, aerospace engineering, and chemical processing. As a major drawback for using H_2_ is its low level of explosion limit (4%), therefore, the precise sensing and controlling systems are essential for hydrogen utilization in storage, handling, transportation and any other usages^3,4^.

It is indeed needed to realize the high-sensitive, high-precision, rapid, robust, real-time, online, and long-distance monitoring of hydrogen concentration. Recently, active and vibrant research activities are involved in the development of various hydrogen sensors^5^. Micro electro mechanical systems (MEMS) based field effect transistor (FET) sensors have more advantages than other conventional sensors, such as small size, low power consumption, intrinsic safety, corrosion resistance, ease of fabrication, high stability, high sensitivity, fast response and recovery time^6^. When hydrogen molecules adsorb and dissociate on the catalytic layer, the difference in work function between the catalytic layer and the semiconductor substrate of the FET changes, which causes a shift in the threshold voltage of the FET^7^. These sensors can detect even a very low concentration of hydrogen gas at a specific position and can be mounted to detect hydrogen gas.

Several types of hydrogen gas sensors based on FET have been studied^8,9^. Tsukada *et al*. developed dual gate FET with Pt-gate and Ti-gate to detect H_2_ gas^10^. The Pt-FET showed a good response to H_2_ gas while the Ti-FET did not show any response to hydrogen gas. Our research group also reported a dual-gate FET H_2_ sensor with Pt-gate by DC/MF sputtering technique^11^. Similarly, the surface of sensing gate was modified with a nano-bumpy shaped Pd film using polystyrene nano-beads as a template to increase the surface area of the catalytic metal film^12^. The surface morphologies of the gate electrode affect H_2_ gas sensing performance. Hence, it is necessary to study in detail the performance of different nanostructures towards detection of H_2_ gas.

FETs with palladium (Pd) gates have been used for H_2_ gas sensor since last two decades. However, a pure Pd metal causes the problem of H_2_ embrittlement and mechanical instability^13^. To overcome these obstacles, alloying of Pd with other nanomaterials offer high catalytic activities. Pd is chosen as the one of the gate element due to its higher hydrogen sensitivity. Silver (Ag) is being a perfect material to combine with Pd for H_2_ gas sensing due to its structural properties and low cost^14^. Addition of Ag to Pd increases the mechanical stability, hardness, chemical inertness and hydrogen permeability^15^. In addition to these, graphene is a two-dimensional (2-D) structure with high carrier mobility, high surface area, and outstanding structural properties. It is a good option for surface modification to gain high sensitivity towards H_2_ detection^16^. The main reason for using graphene_Pd-Ag nanocomposite instead of only Pd-Ag is that the diffusion of H atoms increases along the graphene layer as compared to only Pd-Ag^17^. In addition, the highly conductive nature of graphene enhances the conduction path of the as-proposed sensor. Therefore, graphene with Pd-Ag alloy is a suitable candidate for H_2_ detection due to its high electrical conductivity and large surface area for H_2_ molecular adsorption.

In the present work, nanostructures of graphene-Pd-Ag were deposited on the sensing area to develop the H_2_ gas sensor. A dual-gate FET sensor with sensitive FET was fabricated having a response towards H_2_ while reference FET was not affected by the H_2_ gas. The main aim to use dual-type FETs gas sensor is due to the difference of reference and sensing FETs that affect the threshold voltage offsets because of different gate materials. A multilayer sensor chip was fabricated using a simple MEMS process. To reduce heat loss in the dielectric membrane, an isolated silicon island including the Pt micro-heater was produced using Si bulk micromachining process. To increase the heat efficiency, a Pt microheater was placed on the sensor platform. A micro platform of the multi-layer type structure was fabricated using a MEMS process. The main reason for using graphene decorated Pd-Ag electrode is that decoration of graphene with Pd-Ag nanoparticles would improve the adsorption of hydrogen molecules due to large number of active sites for H_2_ adsorption from Pd/Ag alloy nanoparticles and high carrier mobility of bottom layer graphene. Surface morphologies of the deposited nanostructures were studied. The electrical and thermal characteristics of the FET sensor chip were also measured, and a hydrogen sensing measurement was performed on the fabricated sensor.

## Experimental

### Fabrication of sensor platform

**Figure 1:**
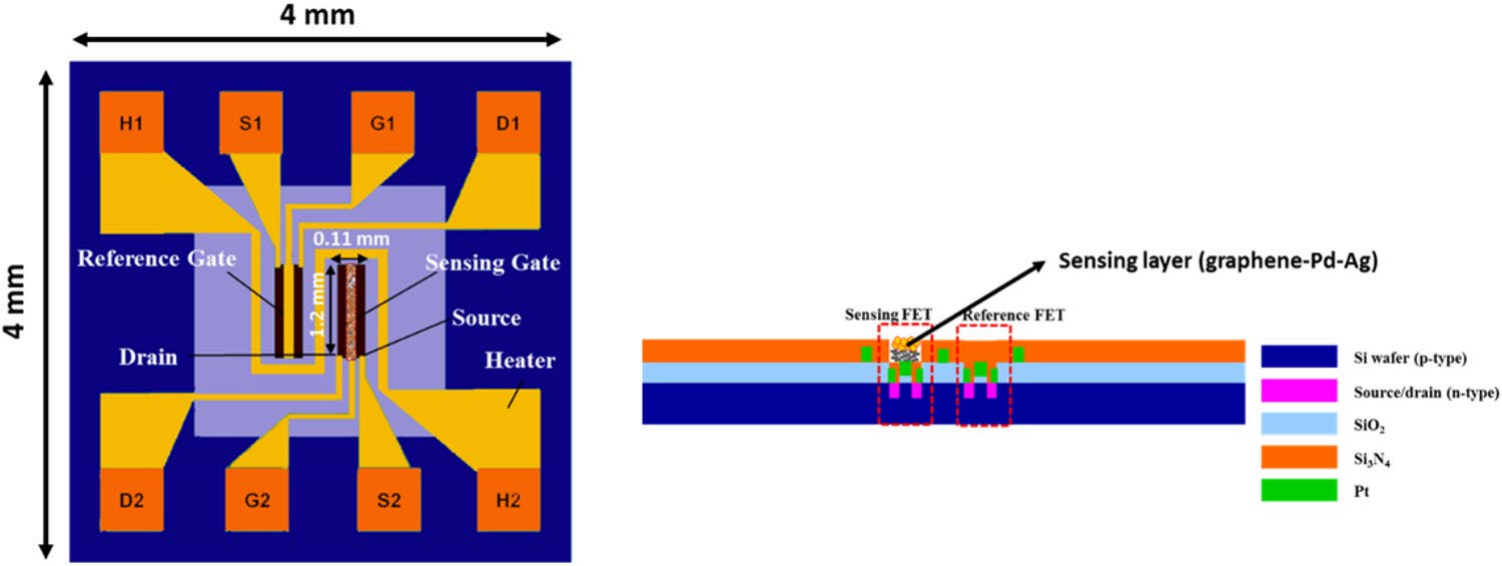
The schematic of hydrogen sensor platform with sensing and reference FETs.

A modified dual-FET sensor platform was designed and fabricated by previously reported method^11^. The schematic of hydrogen sensor platform with sensing and reference FETs is shown in Fig. 1. A micro heater was embedded inside the sensor platform. The sensing-FET, reference-FET and micro-heater were in central area of the platform with the partially isolated membrane. Sensor platform was fabricated with five different photolithography steps. The lithography masks were used for ion implantation and diffusion, an insulation layer (SiO_2_), reference gate and metal contact line (Pt), a passivation layer (Si_3_N_4_), and Si bulk micromachining patterns. The reference and sensing gate electrode pattern (Pt layer), and micro-heater were patterned simultaneously. The back side of the silicon chip was ground by chemical mechanical polishing (CMP) to reduce the thickness up to 100 µm. Finally, sensor platform was diced into the small chip with laser beam cutter. The as proposed H_2_ sensor was outlined with chip dimensions and sensing gate electrode of 4.0 mm × 4.0 mm and 0.11 mm × 1.2 mm, respectively as shown in Fig. 1.

### Surface modification of sensing electrode

**Figure 2:**
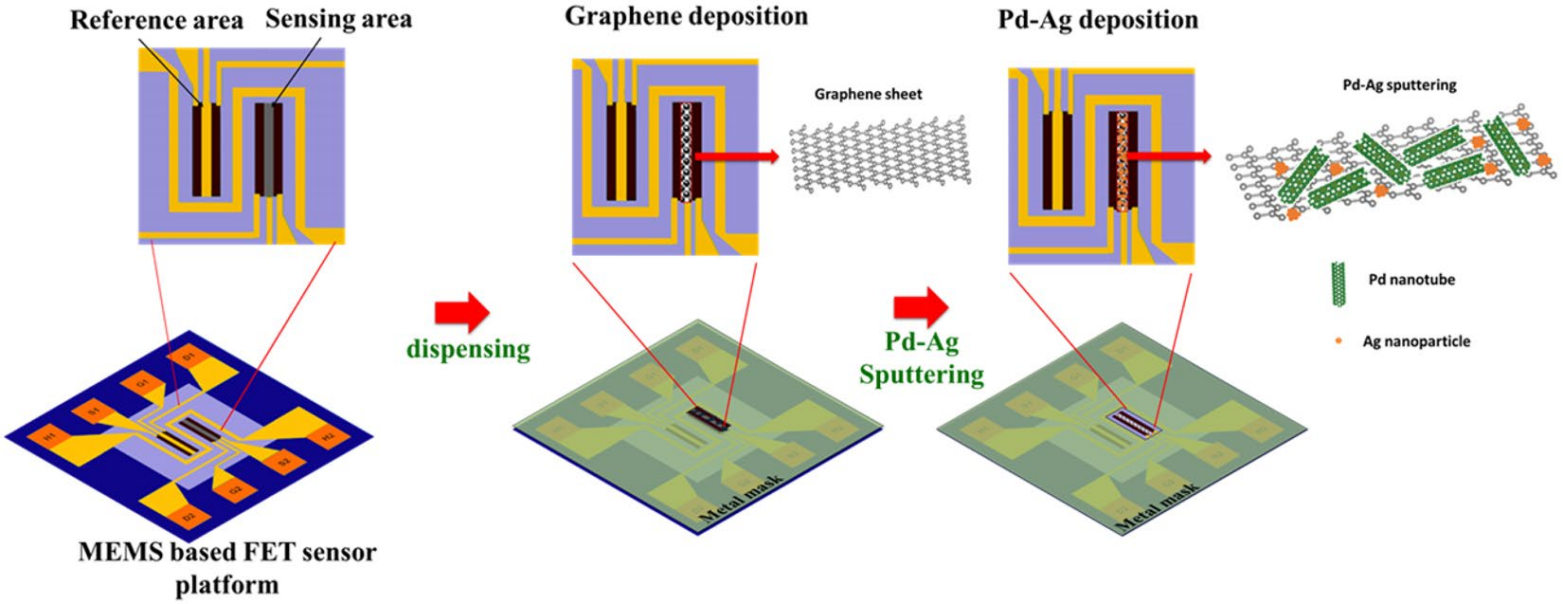
Schematic for the deposition of graphene_Pd-Ag deposition on sensing FET.

The surface of sensing gate electrode was modified with graphene-Pd-Ag nanostructures (Fig. 2). Initially, graphene nanoplates were homogeneously dispersed in an isopropanol solution subjected to ultrasonication for 30 min. The nanoplates dispersed solution was directly deposited onto the sensing FET using a micro dispensing system (MDS 3250^+^, VERMES). Before dispensing, the metal mask was applied on the sensor platform to deposit desired sensing area. The graphene nanoplates were deposited at a constant rate of 0.5 L/min for 1 min. The graphene nanoplates deposited sensor platform was then heat treated at 100 °C for 2 hr under vacuum, following by sputtering of palladium (Pd) and silver (Ag) metal. The thickness of sensing gate electrode was around 30 nm that is controlled by dispensing and sputtering parameters.

## Results and Discussions

### Material characterization

**Figure 3:**
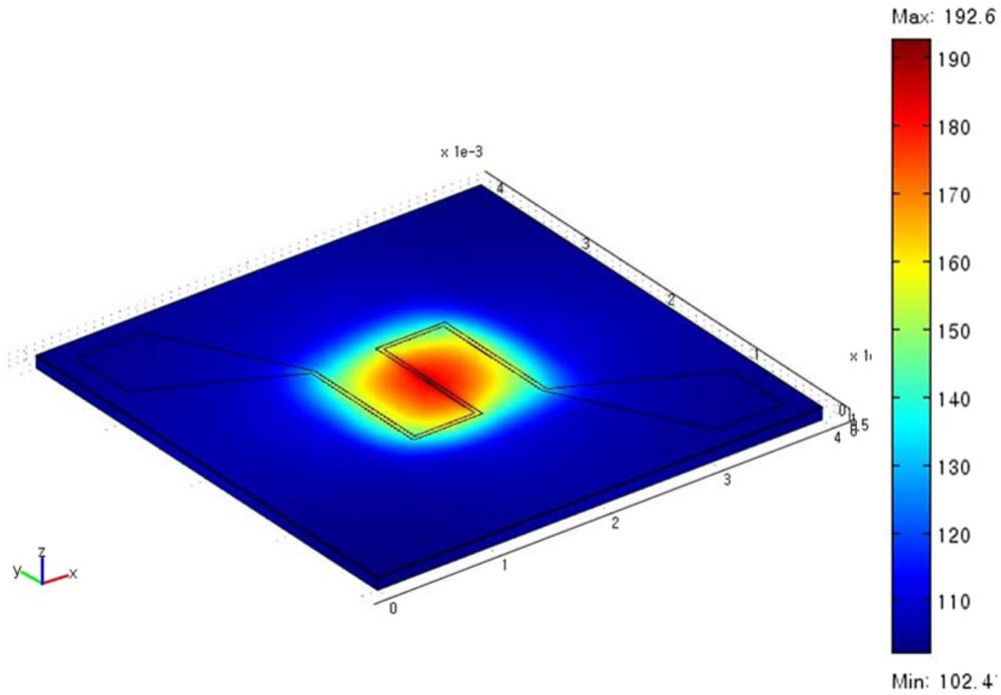
Electro-thermal properties of the GPA-FET embedded micro-heater at 3.0 V.

As shown in Fig. 3, the electro-thermal properties of the micro-heater were analyzed by finite element method (FEM) was analyzed using COMSOL Multiphysics 3.3. Platinum was chosen as the heater element due to its excellent electro-thermal properties. Figure 3 shows temperature distribution in the micro-heater sensor platform at a heater voltage of 3.0 V for the sensor membrane with a thickness of 100 µm. In the present work, the thickness of Si substrate was reduced to be about 100 µm by using CMP micromachining to maintain high temperature distribution at the area of microheater by decreasing the thermal dissipation rate to surroundings^18^. The microheater region was heated homogenously up to high temperature, while the temperature of the outer substrate area was substantially low. The fabrication of microheater near to sensing region can minimize power consumption; provide better temperature uniformity, and low thermal mass. The lowest and highest temperatures of the micro-heater were around 102.4 °C and 192.6 °C respectively. When the heater voltage of 3.0 V was applied, the temperature distribution around the area of the membrane was consistent. The numerical simulation done by FEM explains that the operating temperature of sensing area was controlled well by embedded micro-heater in sensor platform. The main purpose of a microheater is to have high working temperature of the as-formed sensor with low power consumption. The as-proposed H_2_ sensor shows low power consumption of 45.4 mW at 150 °C, suggesting that the H_2_ sensor design can attain a thermal isolation with less heat loss by chemical mechanical polishing (CMP). Accurate temperature control of the sensing electrode and the power consumption of proposed H_2_ sensor was attained by micro-heater operation^19^.

**Figure 4:**
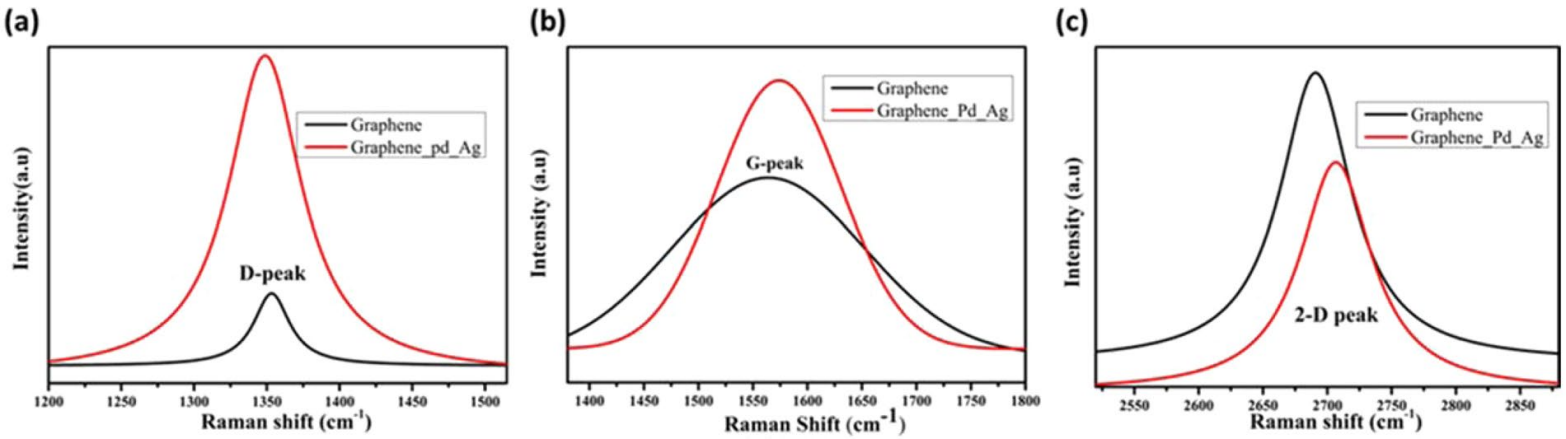
Comparison of Raman spectra for graphene and graphene-Pd-Ag (**a**) magnified view for D-peak, (**b**) G-peak, and (**c**) 2D-peak.

Raman spectroscopy is a powerful tool for studying the structural properties of the carbon structures. The graphene and graphene-Pd/Ag nanocomposites were investigated to detect the doping effects. In Raman spectroscopy, the G and 2D bands occur due to the in-plane bond stretching of the C–C sp^2^ bond, although D bands are related to the different types of defects such as sp^3^ defects that arise from grain boundary edges, electron doping etc.^20,21^. In the present study, the D, G, and 2D peaks are compared between graphene and graphene-Pd/Ag nanocomposites as shown in Fig. 4.

The position of D-peak is changed slightly from 1352.9 cm^−1^ to 1349.5 cm^−1^, whereas the position of G-peak was shifted from 1577.8 to 1563.48 cm^−1^; the 2D-peak is also shifted from 2690.3 to 2706.04 cm^−1^ in the case of the graphene-Pd/Ag to the pristine graphene. The intensity of D peak is increased by 9–10 times in case of the graphene-Pd/Ag as compared to graphene due to more defects and reduces the size of the in-plane sp^2^ domains and the partially ordered crystal structure. The G peak is shifted due to the interaction between the Pd/Ag and the graphene matrix, as it resembles the graphitic nature of graphene. The deviation in G-peak proposes that charge impurities on surface cause electron donating molecules and heterogeneous charge distribution ratios decrease due to doping effect^22^. Furthermore, the downshift of G-peak and upshift of 2D Raman shift can be experienced due to the dynamic effect of carrier population and electrical gating.

**Figure 5:**
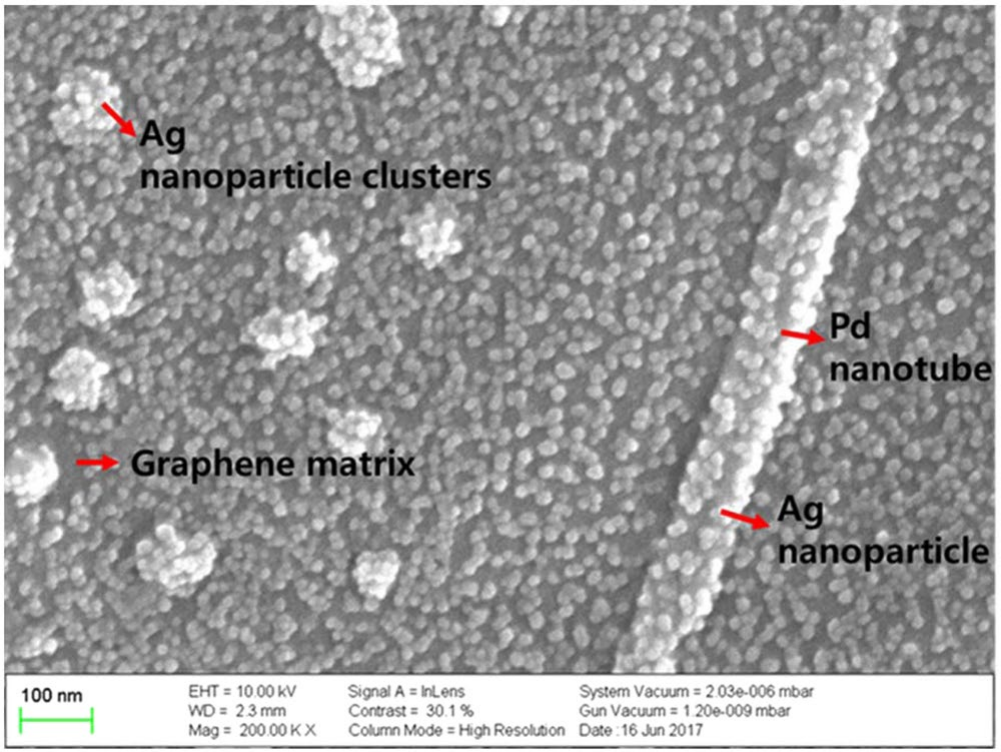
FE**-**SEM images of graphene-Pd-Ag on sensing FETs.

Figure 5 shows the SEM image of the graphene-Ag-Pd nanocomposites on the sensing area of the sensor platform. The integration between graphene, Pd and Ag can be visualized from the SEM image. Ag nanoparticles with the size of ca. 17 nm and Pd nanoparticles at the size of ca. 100 nm are uniformly and compactly embedded on the graphene layer. The average diameter of the Pd nanorods is 65 nm with an average length of 700 nm.

The morphology and nature of the Pd-Ag films grown on the graphene substrate were shown in Fig. 5. Palladium sputtered on the graphene substrate exhibited tube-like morphology. Meanwhile, spherical morphology with few clusters was observed for silver sputtered after palladium. In physical vapor deposition techniques, the morphology and the structure of the thin films are decided by the nucleation and the epitaxial growth which in turn depends on the substrate-adatom interaction^23^. In this case, substrate nature corresponding to the orientation, presence of defects, strain and interface bonding influences the substrate-adatom^23,24^.

In the present work, the graphene used as the substrate had defect peak as shown in Raman spectrograph (as shown in Fig. 4). Major structural defects in graphene correspond to Stone-Wales defects, single vacancy defects, multiple vacancy defects, line defects and carbon adatoms^25^. Defects in graphene can be exploited as the active sites for the nucleation and the growth of metal clusters due to its strong tendency to pin the adatoms^26^. In addition, the nanostructures vary with respect to the mobility of metal adatoms as well as the type of defect on which it is nucleating^27,28^. For instance, line defects like step edges act as an active site for metal adsorption and can be used as a nucleating site for one-dimensional nanostructures^29^. It was reported that palladium atomic diffusion is higher than silver and cesium along the carbon-rich SiC grain boundaries^30^. Hence palladium adatoms are either directly adsorbed on the step edge or adsorbed on the terrace thereby moving towards the step edges due to its high mobility and low free energy on the step edges, attributing to the nucleation and growth of Pd adatoms along the step edges forming Pd nanowires as shown in Fig. 5. whereas silver adatoms sputtered after palladium form in three-dimensional (3D) metal islands (Volmer–Weber growth mode) resulting in Ag spheres and clusters. Further, the increase in the intensity of defect peak in Pg_Ag coated graphene is evidence that metal nucleation occurs at the defect sites.

### Sensing properties

**Figure 6:**
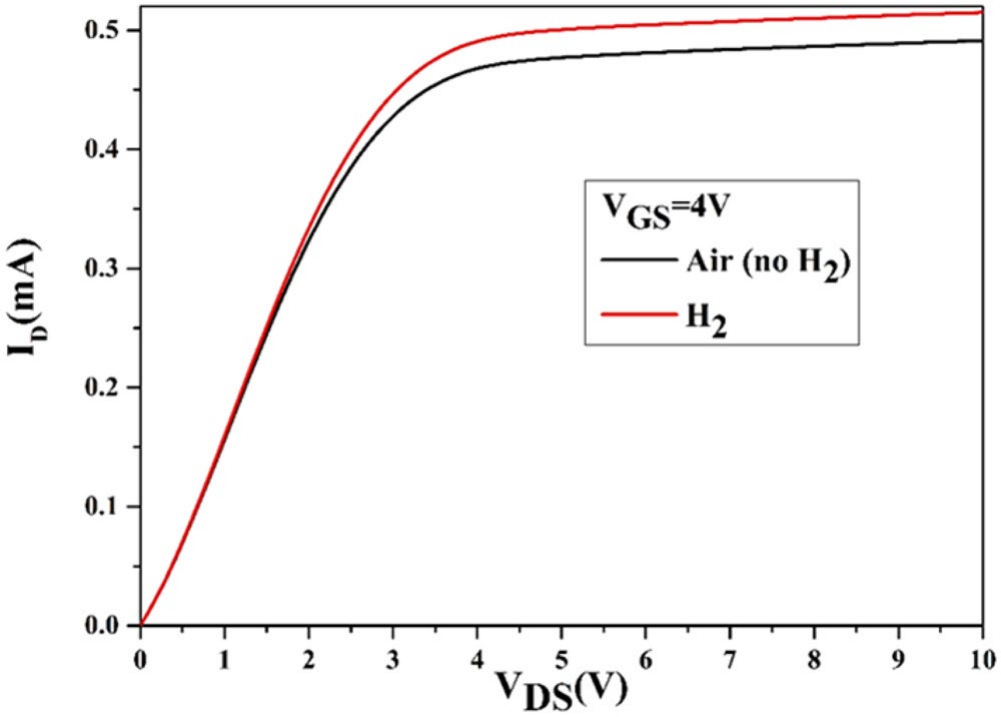
I-V curves are measured from the sensing FET in presence of hydrogen and absence of hydrogen.

The I-V curves were measured for the sensing FET as shown in Fig. 6. In both cases (in presence of hydrogen and absence of hydrogen) the drain current increases linearly and then reach to saturation with increasing voltage^31^. The interaction between H_2_ gas with the graphene-Pd/Ag gate electrode causes the variation in gate potential that results in the variation in the channel width between source and drain^32^. The drain voltage at which the saturation starts is known as pinch-off voltage (V_po_). In both cases (presence of hydrogen and absence of hydrogen), the V_po_ are 4.5 V and 2.85 V and the corresponding saturation currents are 4.86 mA and 4.06 mA, respectively. In hydrogen atmosphere, the V_po_ and I_Dsat_ increased as the channel width increased.

**Figure 7:**
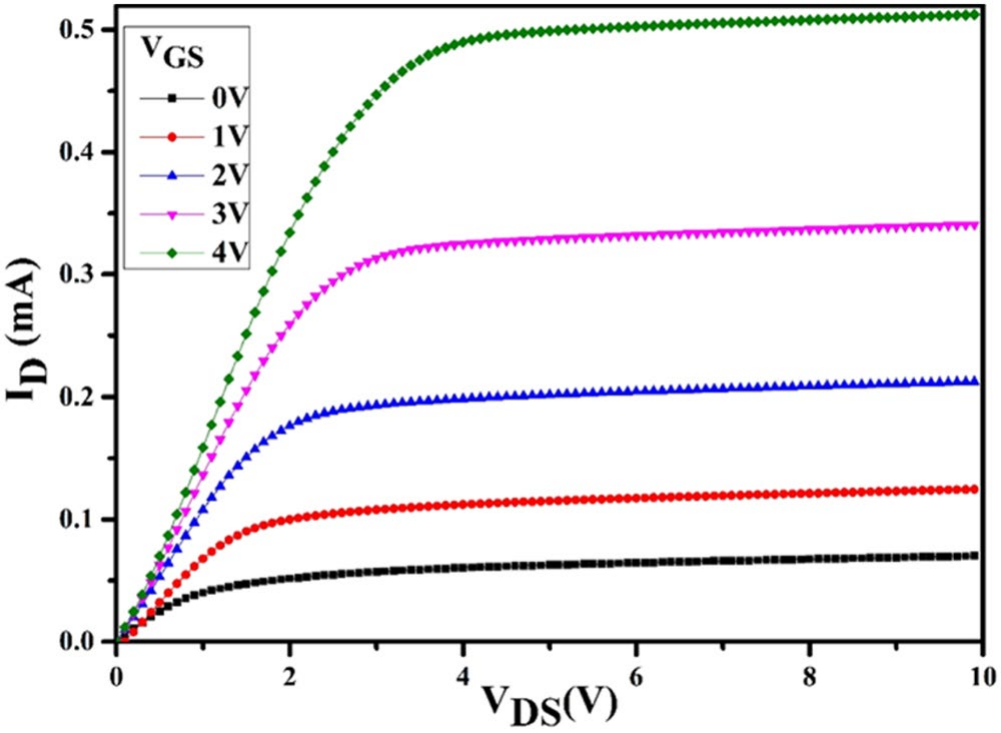
Transfer characteristics of the sensing FET sensor with surface modified with graphene_Pd-Ag gate thin film.

The electrical properties (transfer characteristics) of the as-formed FET sensors are studied as shown in Fig. 7. The transfer characteristic plots the output drain current as the function of input gate bias, for constant drain bias. By increasing the drain voltage (V_DS_), the drain current (I_D_) increases linearly and then saturate. At specific, the saturated drain current (I_Dsat_) is 0.48 mA at 4.0 V V_GS_. By increasing the drain bias beyond the (V_DSAT_) results in the pinch-off to travel more into the channel, closer to the source end. Electrons in the channel are pulled into pinch-off region and travel at the saturation drift velocity due to the high longitudinal electric field along the channel that results in the saturation of drain current^33^.

**Figure 8:**
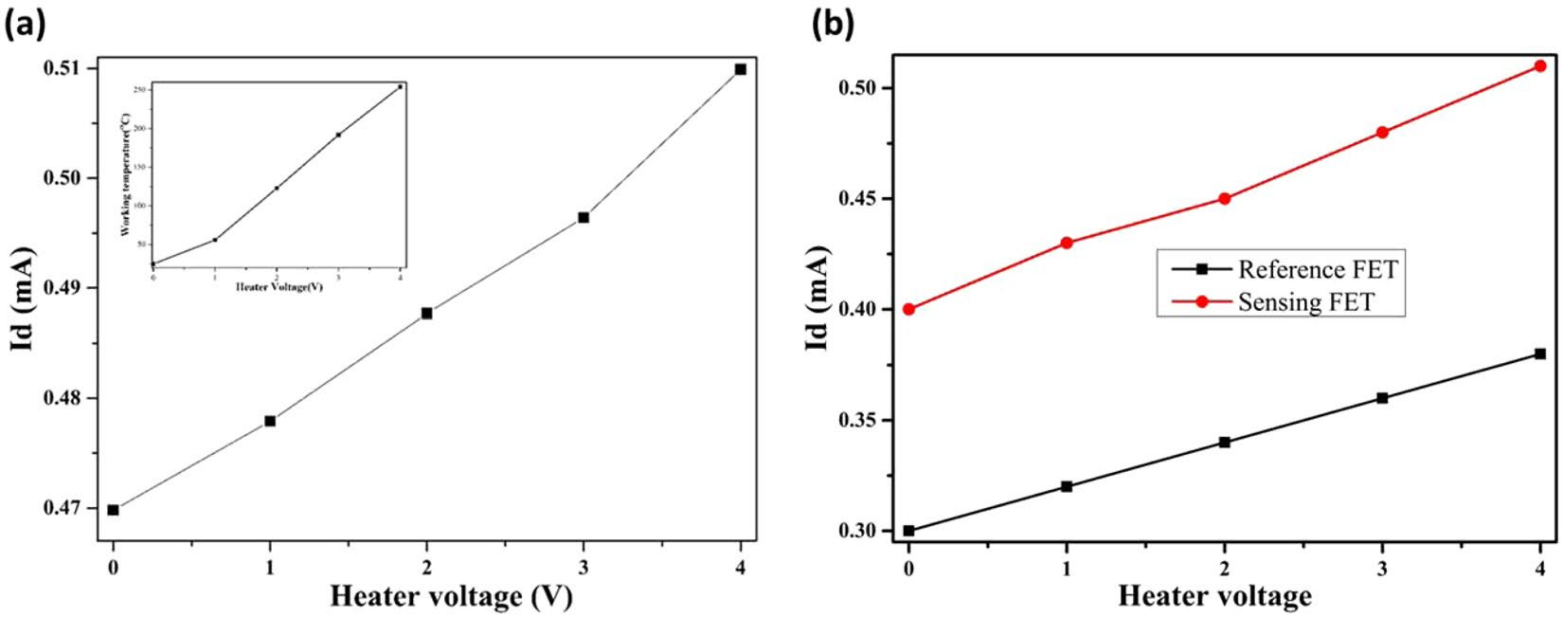
(**a**) Variation in drain current with working temperature. (Inset: relationship between heater voltage and working temperature for sensing FETs) and (**b**) Variation in drain current with sensing and reference FETs.

As mentioned in several reports^34,35^, the drain current changes very slightly with temperature. Figure 8 shows the between drain current and heater voltage and its inset graph show the relationship between heater voltage and working temperature. In inset Fig. 8a, the temperature of as-formed sensor was controlled by using an embedded microheater on the sensor platform. The working temperature of the as-proposed sensors was measured by fluctuating the heater voltages. For heater voltages of 1.0, 2.0, and 3.0, the measured data of temperature are 56.4, 123.6, and 192.5 °C whereas for simulated data is 62.6, 124.7, and 186.7 °C, respectively. In both cases, the working temperature of the as-formed sensor is positively related to the heater voltages. On increasing the voltage of an embedded micro-heater in the sensor platform, there is a slight deviation in the drain current I_D_ due to the change in threshold voltage. Variation in drain current (I_D_) with temperature can be signified as the temperature dependence of charge carrier density, carrier mobility, and threshold voltage^36^. The drain current increases with increasing temperature since the charge carrier density in channel between source and drain increases. The charge carrier density increases due to formation more electron-hole pairs by increasing temperature. Moreover, the H_2_ sensing response of as-formed FET sensor is increasing with increase in temperature due to the hydrogen adsorption process of the as-formed FET sensor is an endothermic reaction. At high temperatures, H_2_ atoms will gain much high energy to facilitate the surface chemical reaction like adsorption, dissociation, and diffusion^37^. As shown in Fig. 8b, the drain currents of both sensing and reference FETs varied by changing the heater voltage from 0 to 4 V. But, the difference between the two FETs is almost constant around 0.1. The main advantage of using the differential drain current as output signal is that the effect of temperature variation can be eradicated for the sensors operating at high working temperature^11^.

**Figure 9:**
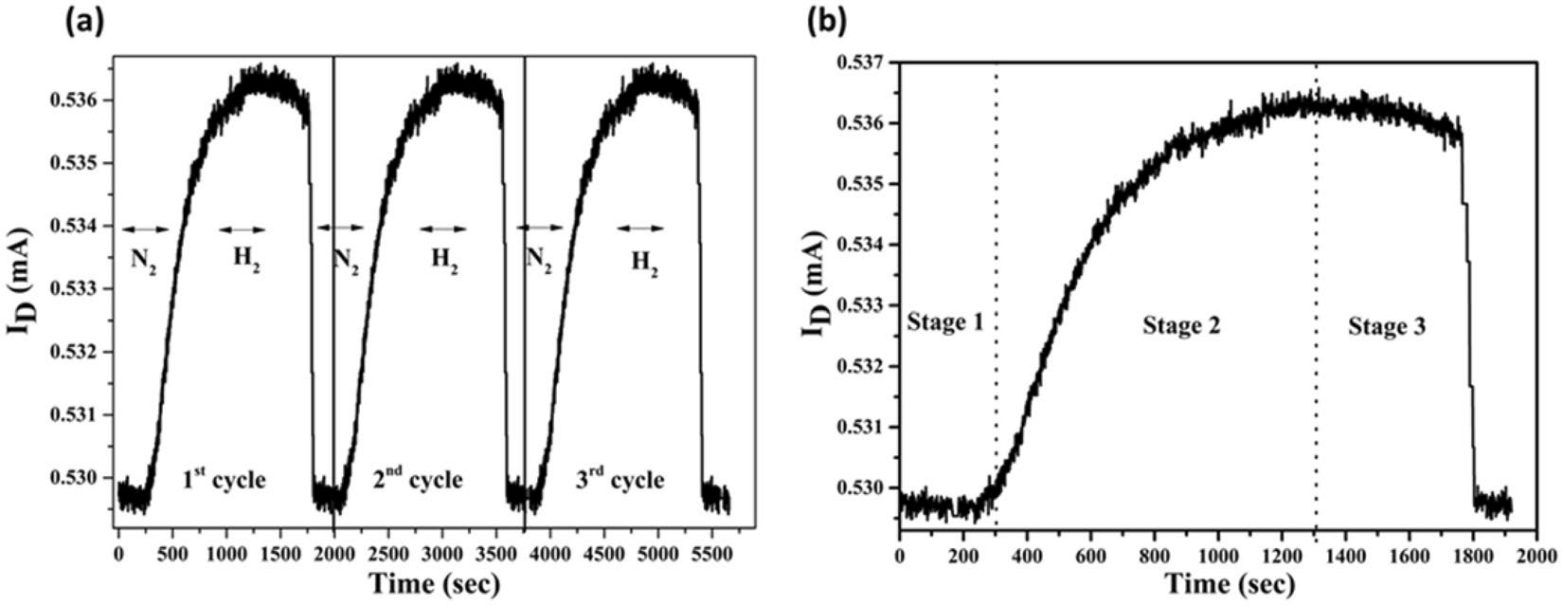
(**a**) shows transient response of the as-formed H_2_ FET sensor at the temperature of 125 °C at the H_2_ concentration of 2000 ppm and (**b**) enlarged view for transient response for H_2_ sensing process.

Figure 9(a) shows the transient response for the as-formed H_2_ FET sensor upon introduction and elimination of hydrogen gas and using nitrogen gas as a carrier gas at the temperature of 125 °C at the H_2_ concentration of 2000 ppm under the applied the drain-source voltage (V_DS_) = 3.8 V and gate-source voltage = 4.0 V. Firstly, the N_2_ gas is put in test chamber to set a drain current baseline of 0.528 mA. By putting the H_2_ gas, the drain current increases rapidly. After few minutes, a quasi-saturation phenomenon becomes deceptive due to dynamic equilibrium between the H_2_ absorption and desorption^38,39^. Under this condition, the maximum variation in drain current was 8 µA. The as-formed FET hydrogen sensors display better stability, even after many testing cycles (Fig. 9). No deviations were detected, and the results attained showed that the drain current was reproducible during H_2_ sensing test. As seen in Table 1, response time and recovery time decrease fast with the operating temperature as hydrogen adsorption/desorption rate increases at high temperature. From the experimental results, the proposed H_2_ sensor displays the best sensing performance at the working temperature of about 245 °C with fast response and recovery time. As shown in Fig. 9(b), the hydrogen sensing process can be divided into 3 stages. Stage 1 is defined as the starting stage. As the H_2_ gas is closed and the carrier gas (N_2_) is introduced, the equilibrium of H_2_ concentration between the Pd/Ag alloy nanoparticles and its oxide is abruptly shattered that leads to the sharp drop in the sensing signal (drain current). After some time, the sensing signal enters stage 2 which defined as the intermediate stage. In this stage, more H_2_ atoms are desorbed due to the fast recombination of H_2_ atoms that leads to the lessen H_2_ atoms on Pd/Ag surface. This may result in fast recovery rate. In stage 3, defined as the final stage, the amount of the absorbed H_2_ atoms on the Pd/Ag surface will increase with time that leads to the increase the binding sites for the H_2_ atoms^40^.

**Table 1:** The Gas sensing properties of the GPA-FET at different operating temperature.

Working temperature (°C)	Response Time (sec)	Recovery time (sec)
RT	68	57
45	59	45
95	47	38
143	35	26
192	28	19
245	16	14

Comparison between response time and recovery time at 1000 ppm of H_2_ gas concentration.

**Figure 10:**
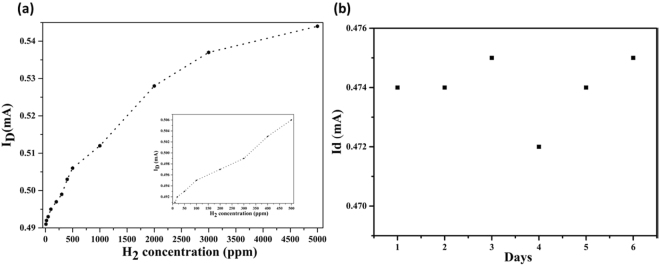
(**a**) Variation in H_2_ concentration with the drain current at a temperature (~125 °C). (Inset: low H_2_ concentration up to 500 ppm.) and Fig. 10 (**b**) long term stability of proposed H_2_ sensor.

The relationship of drain current with hydrogen concentration is shown in Fig. 10, the inset (a) and (b) graph shows long term stability of proposed H_2_ sensor and enlarges data at low hydrogen concentration up to 500 ppm, respectively. It is clear from Fig. 10 that the drain current is directly proportional to the hydrogen concentration. The positive hydrogen concentration dependence of the adsorption reaction rate can be credited to the increased hydrogen adsorption, diffusion, and dissociation coefficients^41^. The drain currents change quickly for all the hydrogen concentrations. The proposed H_2_ FET sensor at a low H_2_ concentration (<10 ppm) showed a good response to hydrogen, therefore the limit of detection (LOD) of the as-formed FET sensor was around 1 ppm. The dual gate-type FET sensors show high response even at low hydrogen concentration below 1 ppm. The high response is due to the interaction between the graphene-Pd/Ag and H_2_ gas. Inset Fig. 10(b) displays the long-term stability of variation in drain current with time. Very slight deviation was found in drain current with time of days at the hydrogen concentration of 1000 ppm. Usually, MEMS based FET gas sensor shows long term stability as compared to other gas sensors^42^. As compared to other reports^43,44^, as-proposed H_2_ sensor shows better sensing properties (fast response and recovery time), the limit of detection (LOD) is around 1 ppm.

**Figure 11:**
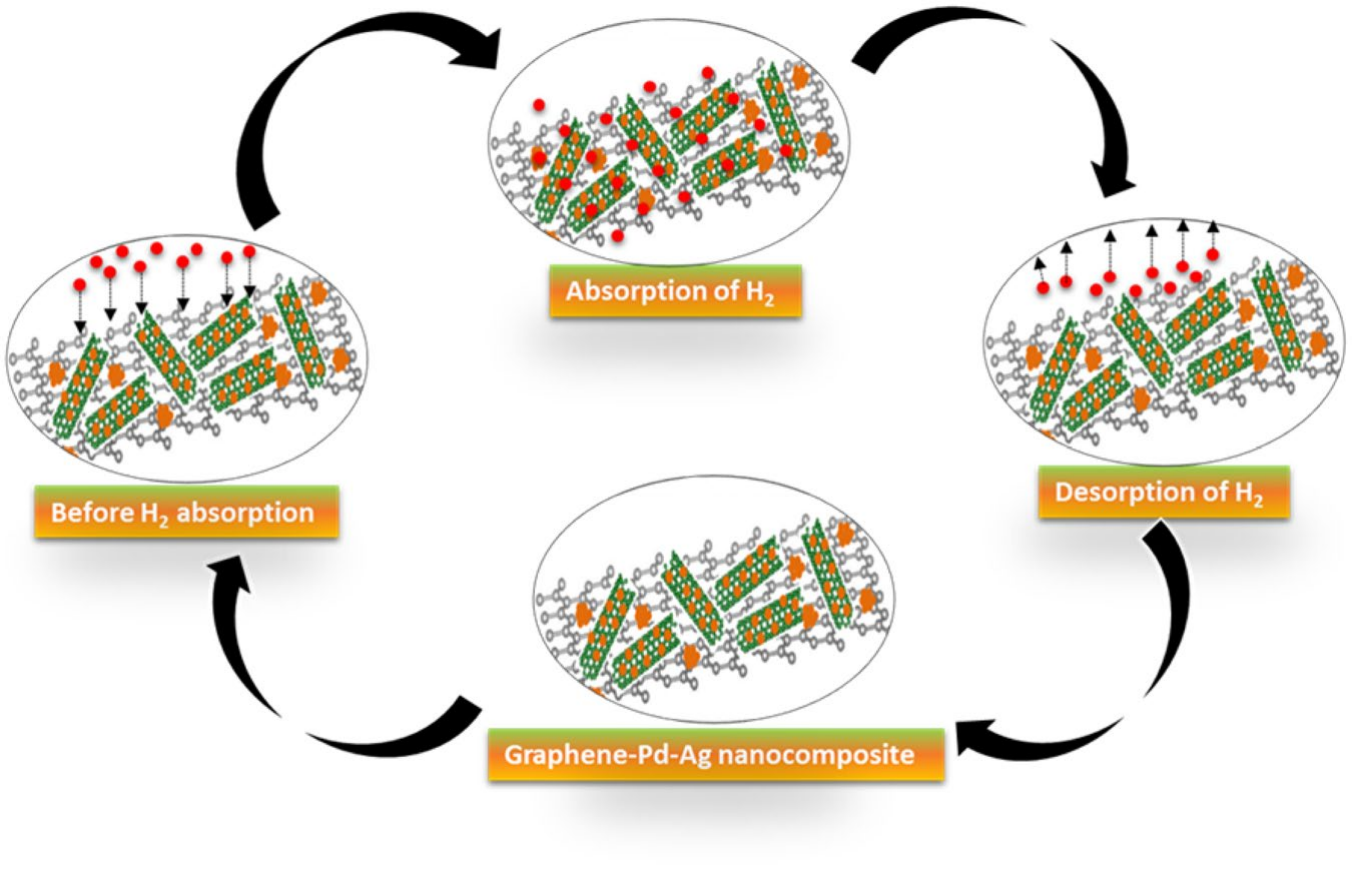
Schematic for H_2_ sensing mechanism in the graphene-Pd-Ag nanocomposite.

Figure 11 is a schematic representation of H_2_ sensing mechanism in the graphene-Pd-Ag nanocomposite. The work principle of the GPA-FET will depend on the difference of work function of the graphene_Pd-Ag (gate electrode) during exposure to H_2_ gas^45^. Initially, the source needs to be grounded then the conductivity between drain and source can be changed by altering the gate voltage (V_GS_). During H_2_ atmosphere, the H_2_ atoms diffuse at the interface between gate electrode (graphene-Pd-Ag) and insulator (SiO_2_) to form a dipole layer which leads to change in gate voltage, which finally results in the change in drain current related to the H_2_ concentration^9^. As H_2_ is a reducing gas and donates electrons to the graphene-Pd-Ag during interaction that leads to the increase in drain current (I_DS_). The increase in the drain current of the graphene- Pd-Ag-gate FET (GPA-FET) can be considered with the process of absorption (physisorption and chemisorption) and desorption taking place on the surface of the graphene-Pd-Ag. With exposure of graphene-Pd-Ag to hydrogen molecules, the formation of metal hydrides takes place that reduces the work function of the Pd-Ag and results in the transfer of an electron from metal hydrides to the graphene^46^. The fast recovery of the as-formed FET sensor is due to the physisorption as it takes place faster than chemisorption. Practically, it is important that the catalytic component (Pd-Ag) is well dispersed in the electrically conductive path (graphene) to maximize the drain current which simply the charge transfer. Figure 11 shows the schematic of the hydrogen absorption and desorption on the surface of the Pd-Ag for the hydrogen sensing mechanism. As shown in Fig. 11, during the H_2_ absorption the H_2_ molecules or atoms are absorbed on the surface of Pd/Ag alloy nanoparticles while in case of H_2_ desorption, all H_2_ molecules or atoms were desorbed from the graphene _Pd-Ag surface.

## Conclusion

A facile method to design and fabricate the dual-gate FET hydrogen gas sensor was developed with embedded microheater. The dual-gate FET hydrogen sensor was integrated with a graphene-Pd-Ag-gate FET (GPA-FET) as sensing FET to detect hydrogen and a Pt-gate FET as a reference one on a single platform. The FEM numerical analysis showed that the operating temperature of sensing area was well controlled by an embedded micro-heater in the sensor platform. The sensing layer of gate electrode was modified with deposition of graphene by an e-spray technique, followed by Pd-Ag sputtering. FESEM results reveal that the graphene-Pd-Ag nanocomposites are uniformly distributed on the sensing electrode and Raman spectroscopy studies the alteration in D, G, and 2D band after the interaction Pd-Ag nanoparticles in graphene and pristine graphene. The GPA-FET showed a good response to H_2_ gas above 1 ppm. The GPA-FET showed a high response to hydrogen gas at temperatures of 25–254.5 °C. The fast response and recovery time were credited to the interaction between the graphene _Pd/Ag nanocomposite and H_2_ molecules. The as-formed FET sensor shows a linear increase in the drain current with H_2_ concentration. The proposed dual-gate FET gas sensor in this study has potential applications in various fields, such as electronic noses and automobiles, owing to its low-power consumption, easy integration, good thermal stability and enhanced hydrogen sensing properties.
